# Novel Fibre-Rich Breads Yield Improved Glucose Release Curves and Are Well Accepted by Children in Primary School Breakfast Clubs

**DOI:** 10.3390/nu17020308

**Published:** 2025-01-16

**Authors:** Nicholas M. Wilkinson, Taskeen Niaz, Eloise Tann, Fiona Croden, Neil B. Boyle, Alan Mackie, Louise Dye

**Affiliations:** 1 School of Food Science and Nutrition, University of Leeds, Leeds LS2 9JT, UK; t.niaz@leeds.ac.uk (T.N.); e.e.tann@leeds.ac.uk (E.T.); a.r.mackie@leeds.ac.uk (A.M.); 2 School of Psychology, University of Leeds, Leeds LS2 9JT, UK; f.c.croden@leeds.ac.uk; 3 School of Psychology, University of Sheffield, Sheffield S10 2TN, UK; n.boyle@sheffield.ac.uk

**Keywords:** fibre, in vitro digestion, school breakfast club, liking, acceptance

## Abstract

Background: The average fibre consumption of 4–10-year-old children in the UK is 14.6 g per day, with only 14% of these children reaching the 20 g recommended by the SACN (UK Scientific Advisory Committee on Nutrition), and this ‘fibre gap’ may be most pronounced in communities with the lowest socioeconomic status. School breakfast clubs target children from disadvantaged communities, but their provision may favour lower-fibre foods, due to perceptions that children will reject higher-fibre foods. Our research programme aims to increase the fibre density, digestive-metabolic quality and acceptability of school breakfast provision. Methods: In Study 1, we examined the in vitro digestion of four novel bread products, to determine the relationship between fibre content and glucose release profile, and assess their suitability for sustaining school activity. In Study 2, we introduced the Prograins breads, alongside higher-fibre breakfast cereals and fresh fruit, to primary school breakfast clubs. Results: The Prograins bread products yielded lower peaks and more sustained glucose release curves than the ‘standard’ white bread control. Many children liked and chose the intervention foods, and the average fibre content of children’s breakfasts increased. Conclusions: We conclude from this study that nutritious, fibre-rich bread products can be acceptable to children and that higher-fibre breakfast provision is feasible, and we recommend larger-scale intervention and assessment to validate these real-world findings.

## 1. Introduction

The importance of dietary fibre for digestive health is well recognised [1]. However, low fibre consumption is endemic and typical in the UK population. In 4–10-year-old children, the average fibre consumption is 14.6 g per day, with only 14% reaching the SACN-recommended 20 g for children within this age group [2]. The Danish Wholegrain Partnership is an example of a successful intervention to increase fibre consumption at the population scale, though this initiative was less successful in increasing fibre intake in groups with the lowest socioeconomic status (SES) [3]. Here, we focus on school-based interventions to increase fibre consumption, including children from low-SES backgrounds. Many UK schools offer breakfast clubs which combine childcare provision and breakfast provision. These schemes are often subsidised for children who are eligible for free school meals, making them a good way to access children from households likely to be facing food insecurity [4]. Significant cognitive and behavioural benefits of school breakfast provision have been reported [5,6], and cost–benefit analysis of school food improvement indicates good long-term value-for-money investment [7]. Thus, targeting school breakfast clubs has the potential to increase children’s fibre consumption in those most at need and contribute to transforming the dietary habits and preferences of the next generation [8]. However, preference (actual and perceived) may be a barrier. Providers and school staff are keen to supply food that children will eat and enjoy, and can be risk-averse in this regard. Here, we examined the extent to which children’s preferences and resistance to dietary change represent a barrier to the introduction and consumption of higher-fibre foods in two primary school breakfast clubs in the Leeds region of the UK.

Responses to our ongoing (2024–5) survey of Leeds schools (170 respondent schools to date) indicate that over 97% of school breakfasts include bread or bagels, with 77% of schools offering refined, white versions at breakfast, and 39 schools (28%) not offering any wholegrain or ‘50:50’ breads alongside. White bread is a widely consumed staple in the UK (including in schools), but is low in fibre [9]. However, white sliced loaf bread still occupies a default and somewhat totemic status in the UK [10], especially in lower-income communities [9]. Higher-fibre breads may therefore be perceived as less familiar and less palatable by children and/or school staff, especially in lower-income communities [8,9,11]. ABMauri has recently developed the ‘Prograins’ range of higher-fibre breads which aim to combine broad appeal with beneficial digestive properties. Fibre content is one of multiple factors (including protein content and fibre type) which can affect the bioavailability and metabolism of glucose in food [12,13], so it is important to look at functional digestive performance as well as calculated fibre content.

Here, we examine whether (a) Prograins breads offer desirable temporal profiles of digestive glucose release, and (b) Prograins breads and other higher-fibre breakfast staples are acceptable to children in primary school breakfast clubs. There is limited research directly measuring children’s responses to tasting bread products. Research on children’s consumption responses to breads has focussed on preference (A vs. B), and on white versus wholemeal bread [14]. Studies have often used visual questionnaires to assess preferences [15,16,17,18], or examined choice and preference in focus groups [19]. Here, we focus instead on real food tasting, on liking/acceptance rather than comparative preference, and on novel fibre-rich bread products rather than standard wholemeal wheat bread. This is because we are interested in whether children will taste, eat, and enjoy the product per se (more than whether they prefer it to another product), and because we want to assess the potential of innovative fibre-rich breads for child nutrition. Further, we want to interrogate the full range of sensory engagement with the foods (not just visual assessment and imagined preference). These factors will directly contribute to the viability of these products in school breakfast clubs.

Digestion of carbohydrate-rich food in the upper gastrointestinal tract (GIT) leads to glucose release, and its subsequent absorption increases postprandial glycaemic index (GI). GI is a measurement of blood glucose response following the ingestion of starchy food [20]. Consumption of food with a high GI results in various hormonal and metabolic changes that are associated with diseases such as type 2 diabetes (T2DM), obesity, insulin resistance (IR), and poorer cognitive function, especially in children [21,22]. In contrast, food with a low GI (slow digestion of starch) can help to control appetite and delay hunger, and hence can effectively reduce the risk of non-communicable diseases (NCDs), e.g., hyperinsulinemia, lipidemia, etc. [22,23]. Due to these health-related benefits, a low-GI diet is considered a potential therapeutic alternative for treating childhood obesity and other NCDs in children and adults [24,25]. Due to the invasive nature of GI measurement in vivo, in vitro digestion models have recently been used to predict the glucose response for different foods, offering lower cost and time requirements, reproducibility, a lack of ethical limitations, and positive correlations with in vivo glycaemic responses [26,27].

In Study 1, we used in vitro digestion [28] to examine the time course of glucose release for four ‘Prograins’ bread products, as well as three ‘standard’ breads for comparison, all supplied by ABMauri. White bread typically releases glucose quickly, providing a rapid high peak followed by a fast drop in gastrointestinal glucose availability [29]. In the context of school breakfast, breads which release glucose more slowly and steadily are more desirable [21], both to minimise spikes in insulin response (which responds to elevated blood glucose on a <10 min timescale [30]), and to provide a continuous steady supply of glucose throughout the ~4 h between breakfast and lunch (when 80% of the school curriculum is taught) to fuel activity and cognition [31]. Therefore, alongside predictions of the overall and 2 h glycaemic index (‘GI’) for the Prograins breads and comparators [32], we report detailed glucose release curves. Individual differences notwithstanding, higher glucose release can be expected to lead, on average, to higher absorption and postprandial glycaemia, and hence higher insulin response [33].

In Study 2, we introduced the Prograins breads, plus fibre-rich breakfast cereals, to two primary school breakfast clubs. First, we assessed children’s liking for the breads and cereals using a simple taste-and-rate activity during breakfast club time, but distinctly from breakfast consumption. Then, we observed take-up levels for intervention foods when offered to the children alongside the usual breakfast club provision. We also provided fresh fruit and examined take-up. We did not conduct preliminary tasting, nor measure liking, for the fresh fruit. We wanted to know the extent to which children would (a) like/dislike, and (b) choose the higher-fibre foods, following the opportunity to taste them first. This will inform us as to whether this approach is feasible for interventions in school breakfast clubs. Here, we report quantitative data and qualitative narratives from the children in response to the breads to motivate and inform recommendations for larger-scale interventions and outcome assessments in future work.

## 2. Materials and Methods

### 2.1. Study 1: In Vitro Digestion of Breads

#### 2.1.1. Materials—Study 1

Bread samples with different fibre concentrations were provided by AB-Mauri; α-amylase from human saliva (A1031), pepsin from porcine gastric mucosa (P7012), pancreatin from porcine pancreas (P7545), and bile extract porcine (B8631) were purchased from Sigma Aldrich (St. Louise, MO, USA). A Total Starch Assay Kit (AA/AMG) for glucose detection was purchased from NeoGen/Megazyme (Bray, Ireland). Analytical-grade HCl (97%), NaOH (1 M), and all salts used to prepare electrolyte stock solutions were also purchased from Sigma Aldrich. All solutions were prepared with milli-Q water, with a resistivity of 18.2 MΩ cm, at 25 °C (Milli-Q apparatus, Millipore, Bedford, UK).

#### 2.1.2. In Vitro Gastrointestinal Digestion

Four bread samples: sprouted multispeed bread (SMB), Heritage Spelt (HSB), BARLEYmax^®^ (BMB), Protein power bread (PPB); and four control bread samples: standard white bread (SWB), standard 50% (S50), and standard wholemeal (SWM), were subjected to simulated GI digestion. Twenty grams of each bread sample was digested. Oral and gastric in vitro digestions were performed by the INFOGEST semi-dynamic in vitro model, as described in [34]. See the Supplementary Materials for full details of this protocol as followed in this study.

#### 2.1.3. Sampling and Glucose Detection

**Figure 1:**
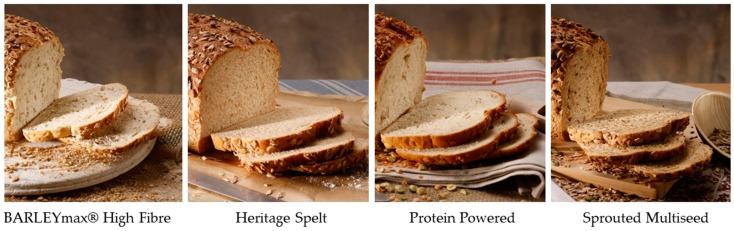
Visual aspect of the four novel bread products.

During intestinal digestion, 500 μL of digested samples were taken at 0, 10, 30, 60, 90 and 120 min. Thus, there were 30 time point samples for each complete digestion as shown in Figure 1 for the standard white bread. For the determination of glucose Pefabloc (5 mmol/L final concentration) was added to each sample to stop pancreatin enzyme activity and samples were placed in a water bath (100 °C for 5 min) to stop amylase activity. After 5 min samples were stored at −80 °C for further analysis. Digesta samples were then analysed for their bioaccessible glucose concentration using a standard total starch kit (Megazyme K-TSTA-100A) method E (a) and the amount of glucose released was calculated with reference to glucose standard. Briefly, the first step with α-amylase digestion was skipped because the samples had already been digested. Amyloglucosidase was used to convert polysaccharides into monosaccharide glucose units (detailed protocol available in the Supplementary Materials). Finally, GOPOD reagent was added into each sample and incubate the solutions at 50 °C for 20 min and measure absorbance against the reagent blank at 510 nm.

### 2.2. Study 2: Acceptability in School Breakfast Clubs: A Feasibility Study

#### 2.2.1. Materials—Study 2

Breads/toast: Four novel ‘Prograins’ bread products, provided by ABMauri, were used. See Figure 1 for the visual aspect. ABMauri also supplied three ‘standard’ breads (white, wholemeal, and combined white–wholemeal). We refer also to the Warburtons half&half^®^ bread that is typically provided in Magic Breakfast clubs, which contains minimal particulate ‘bits’ to appeal to people accustomed to white bread. We refer to this combined white–wholemeal bread type as ‘50%’ bread.

1. A ‘BARLEYmax^®^ High Fibre’ bread, very high in fibre (3.97 g/slice, 11.3 g/100 g), but with a light visual aspect. Somewhat ‘bitty’ due to barley flakes.
2. A ‘Heritage Spelt’ bread with no bits/seeds. Source of fibre (1.44 g/slice, 4.1 g/100 g).
3. A ‘Protein Powered’ bread fortified with legumes, close to white and with only very small visible ‘bits’. Source of fibre (2.03 g/slice, 5.8 g/100 g).
4. A ‘Sprouted Multiseed’ bread, high in fibre (2.35 g/slice, 6.7 g/100 g), and seeded, with a darker visual aspect.

High-Fibre Breakfast cereals: Three intervention breakfast cereals made by Kellogg’s were used. The Rice Krispies multigrain products include wholegrain fibre and inulin, and the Raisin Wheats include fibre from wholegrain wheat and dried fruit. These products are relatively high in fibre and relatively low in sugar in terms of the breakfast cereal market.

1. Rice Krispies Multigrain Shapes (Strawberry and Apple)—a 30 g serving has 4.8 g of fibre and 2.3 g of sugars
2. Rice Krispies Multigrain Shapes (Honey)—a 30 g serving has 3.6 g of fibre and 3.0 g of sugars
3. Raisin Wheats—a 30 g serving has 2.7 g of fibre and 3.9 g of sugars

Low-fibre breakfast cereals ‘enhanced’ with porridge oats and strawberries: We added porridge oats and strawberries to the two low-fibre cereals served in Magic Breakfast clubs, Cornflakes and Rice Krispies. We estimated this augmented cereal to have 2.5 g of fibre per serving; 20 g of Cornflakes/RiceKrispies (mean fibre 0.43 g), plus 20 g of oats (~1.8 g fibre), plus 15 g of strawberries (0.3 g fibre), giving a total of 2.53 g of fibre, rounded to 2.5 g.

#### 2.2.2. Methods

Design: A cross-sectional study of food acceptability/liking in primary school children.

Participants: Children aged 4–11 years old participated in the taste tests during their attendance at two school breakfast clubs. The data collection was anonymous, and gender was not recorded. Attendance was recorded each day, but individuals were not identified. The mean attendance in School 1 was 32.8 children per day, and 33.3 children per day in School 2, with an approximately 50/50 male/female ratio in each school.

Ethics and recruitment: The study received ethical approval from the University of Leeds School of Psychology Research Ethics Committee, reference PSYC-896, on 12 May 2023. Caregivers were informed in advance and given the opportunity to opt out their child. One caregiver–child dyad opted out.

Intervention: We examined the acceptability of test foods in two ways: a taste-and-rate activity, and as part of breakfast service. These activities were conducted in two Magic Breakfast clubs in two primary schools in Leeds, UK.

Taste-and-rate activity: During the school breakfast club, but separately from their meal, children were invited to taste and rate food samples on a 3-point scale with the categories ‘Yuk’, ‘Ok’, and ‘Yum’. Ratings were made by placing the food sample container on a visual–verbal placemat presenting ‘emoji’-style faces alongside these rating words. The children’s comments were also noted.

Breakfast service: Alongside the standard Magic Breakfast food provision, we added a set of test foods. Children could choose any of the standard and/or new test foods. We recorded breakfast choice anonymously. Many children visited the breakfast service buffet more than once, and these multiple visits were not attributable to individuals. The data are therefore presented at the group level.

**Figure 2:**
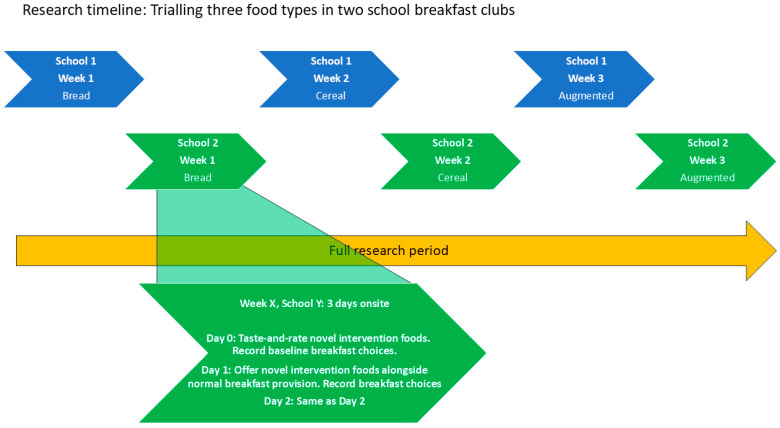
Research timeline; six weeks in two school breakfast clubs, testing three food categories in each school.

Research timeline: We examined responses to 3 sets of intervention foods. Each set of foods was offered separately, over successive weeks. In week 1, we offered four novel breads (toasted). In week 2, we offered three breakfast cereals. In week 3, we offered the low-fibre cereals in the normal breakfast club offer (Cornflakes and Rice Krispies), but with added porridge oats and strawberries, which we label ‘Augmented’ cereals. For each research session, we collected data over 3 consecutive days (Tuesday–Thursday). We recorded children’s breakfast choices on all three days, using Day 0 as a baseline with the standard breakfast club menu. Children could opt into the taste-and-rate activity on Day 0. On intervention Day 1 and Day 2, we offered the ‘breakfast service’ aspect. Alongside all the food sample types, we offered fresh fruit with the breakfast service. For weeks 1 and 2 this was whole fruit, and for week 3 it was cut fruit. We did not conduct the taste-and-rate activity for fruit. See Figure 2.

Why taste-and-rate? We offered a taste-and-rate activity for novel foods during play-time in the breakfast club, separately from breakfast consumption and presented as a test of the food, not of the children. Providing the opportunity to taste enables children to assess novel items and hence make informed breakfast choices. Exploratory tasting activities are acceptable to many primary school children [35], and are favourably viewed by children and parents in focus groups as a mode of introduction for novel foods [19]. This may help to avoid neophobic responses in some children. The taste-and-rate activity also provided useful information regarding preferences for the various products, which may also be useful for breakfast club staff and providers. Some breakfast club staff we worked with already used a version of this simple but effective technique for introducing new foods.

Data collection: At baseline, we collected liking data from the taste-and-rate activity, plus baseline breakfast choice data (i.e., normal breakfast club with no intervention foods). On intervention Day 1 and Day 2, we collected breakfast choice data with the intervention foods on offer. This process was repeated over the three intervention weeks, with the three sets of intervention foods. See Figure 1.

Measures: Each child that chose to participate in the taste-and-rate activity rated each chosen food sample (Yum, Ok, Yuk), and we report these ratings directly. To evaluate children’s willingness to choose higher-fibre foods during breakfast service, we recorded all food choices, and calculated the mean number of food items chosen per child attending the breakfast club. We graphically present food choices in terms of the contribution of each food to the fibre content of the average (per child) breakfast chosen (this measure is of the food chosen, and does not account for uneaten/waste food).

Wastage: We were unable to accurately measure wastage due to the use of numerous waste routes, not all of which were within the breakfast club area. However, we observed wastage, noting any food items yielding particularly high wastage (e.g., half-eaten or in the bins).

Fieldwork Background: Magic Breakfast is a school breakfast club provider/supplier. We worked with Magic Breakfast to conduct this research in schools that they supply. Magic Breakfast is registered under charity number 1102510 in England and SC048202 in Scotland. In co-incidence with this study, Magic Breakfast introduced a new breakfast range with increased fibre content. Therefore, the baseline fibre density of the standard breakfast offer was quite high.

## 3. Results

### 3.1. Study 1: In Vitro Digestion

**Figure 3:**
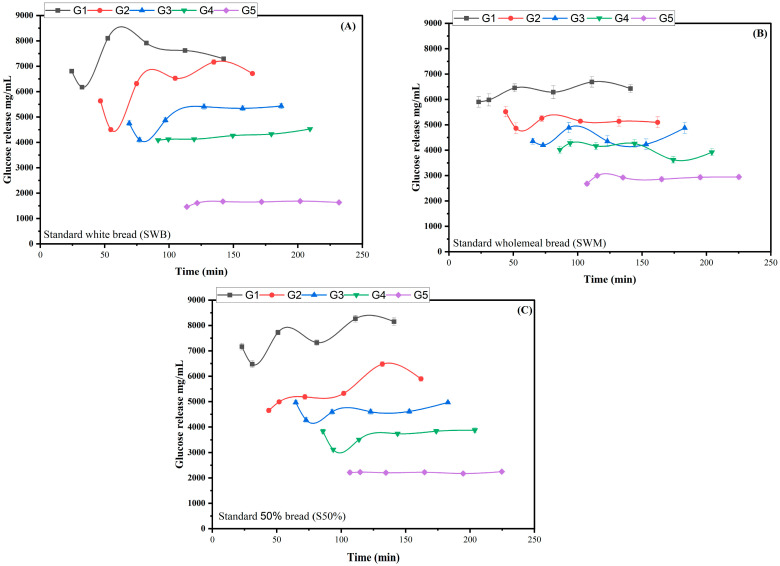
Glucose release after semi-dynamic digestion of standard bread samples. G1, G2, G3, G4, and G5 represents five gastric emptying points. (**A**) standard white bread, (**B**) standard wholemeal bread, and (**C**) standard S50% bread.

Starch digestion occurs primarily in the small intestine. However, if the gastric pH remains relatively high, salivary amylase will remain active in the gastric phase. This is exemplified in Figure 3A, which shows that the starch in standard white bread is soon digested, with little remaining at the end of the gastric phase (G5). Only if there is relatively little gastric starch digestion, and access to the remaining starch is limited, will the starch continue to be hydrolysed over the course of the intestinal phase of digestion, as shown for G5 in Figure 3A.

**Figure 4:**
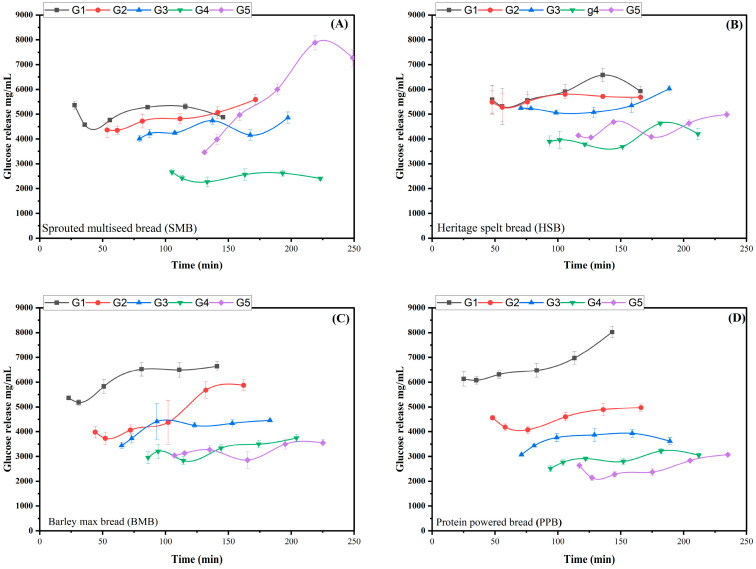
Glucose release for Prograins breads after semi-dynamic digestion. G1, G2, G3, G4, and G5 represent five gastric emptying points. (**A**) Sprouted Multiseed bread, (**B**) Heritage spelt bread, (**C**) BARLEYmax^®^ bread, and (**D**) Protein Powered bread.

**Figure 5:**
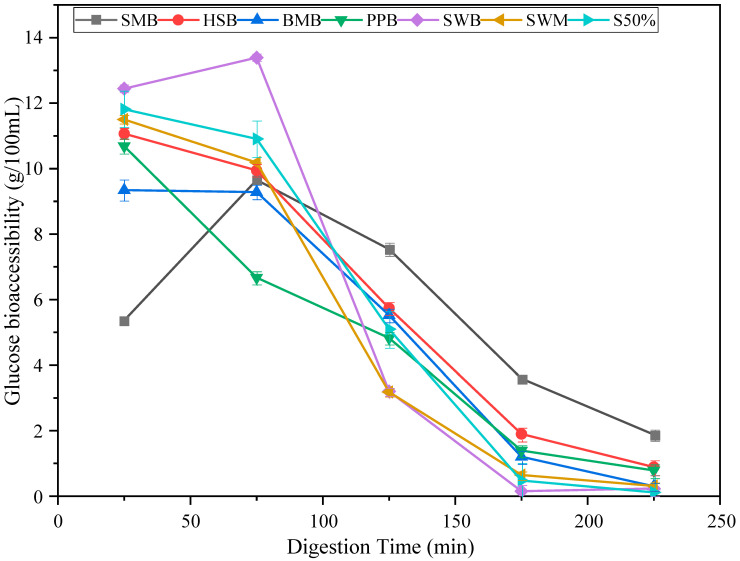
Predicted absorbed glucose as a function of time for the four Prograins bread samples and three standard bread samples. Data are mean (*n* = 3) ± standard deviation (SD). SWB = standard white bread. S50% = standard 50% bread. SWM = standard wholemeal bread. SMB = Sprouted Multiseed bread. HSB = Heritage Spelt bread. BMB = BARLEYmax^®^ bread. PPB = Protein Powered bread.

The data displayed in Figure 3 and Figure 4 are difficult to compare to standard plasma data because of the five parallel intestinal phases. Therefore, to make the in vitro data comparable with in vivo data, we summed specific time points into time bins, and assumed that any glucose was absorbed rather than accumulated, to generate bioaccessible glucose curves as a function of time. The time bins we have used are for every 50 min, so the first data point includes the first intestinal glucose concentration, plus any increments in concentration that occurred in any of the intestinal samples taken before 50 min. If there was no increment or a decrease in intestinal phase glucose concentration, we assumed the value to be zero. Thus, for the white bread, the data at 50 min comprises the sum of the first concentrations from G1 and G2. As the concentration of the second data point in G1 decreased, this value was set to zero. The second bin is thus for 50 to 100 min, and includes the increment in glucose from G1 or G2, plus the initial concentrations from G3 and G4. This approach was used for all the data, and is shown in Figure 5 for all four samples and the three standard breads. As expected from Figure 3A, the standard white bread had the fastest predicted glucose absorption.

The data shown in Figure 5 should be interpreted by comparing them with the standard white bread. As expected, the standard white bread shows the fastest release of absorbable glucose, and consequently the fastest drop in glucose. This contrasts with the delayed glucose curve for the SMB. The delayed glucose curve for the SMB is caused by two main factors. Firstly, the energy density is higher, and so the gastric emptying rate is slower, at 131 min versus 114 min for the SWB. Secondly, there is a more persistent structure in the gastric chyme that also contributes to the slower release of glucose. The Heritage Spelt and the BARLEYmax^®^ breads yield slightly flatter curves than the standard wholemeal, whilst the standard 50% bread is about half-way between the standard white and standard wholemeal. The Protein Powered bread yields notably less starch than the other bread samples overall.

**Figure 6:**
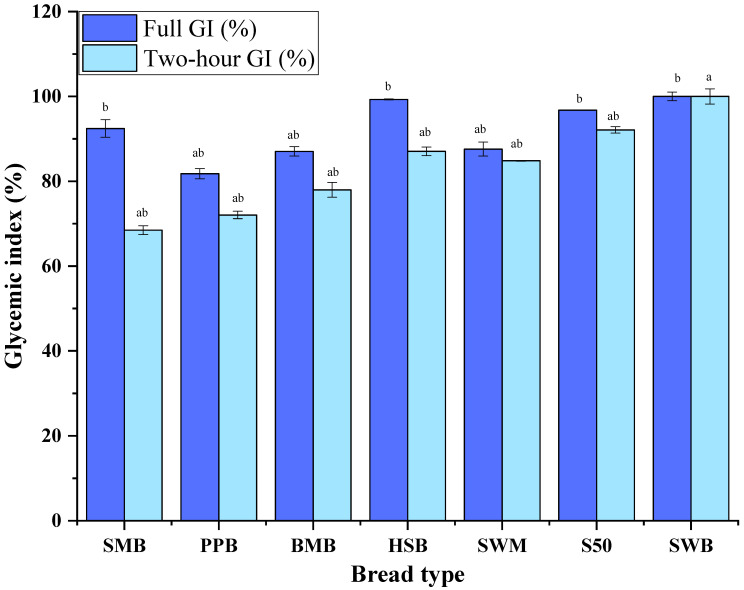
Simulated glycaemic index (area under accessible glucose curve). Values are means *n* = 3 ± SD (single letters ‘a’ and ‘b’ represent non-significant difference, and ‘ab’ represents significant difference with *p* < 0.05).

The data in Figure 5 can be used to calculate the area under the curve (AUC), which can then be normalised against the value for white bread. The AUC was calculated for the full curve or for the first 2 h to calculate the predicted glycaemic index (GI); the results are shown in Figure 6. It should be noted that the full AUC values did not indicate delayed glucose accessibility, as seen for the SMB, for example. It has been observed previously that glucose response should be observed at the beginning of digestion, which is more critical since fast starch digestion is linked to this stage [36,37,38]. Therefore, a better measure for this is the 2 h GI.

The 2 h GI decreased in the following order: SMB < PPB < BMB < SWM < HSB < S50 < SWB (Figure 6). This could be attributed to the different composition of the bread types. Similarly, the lower GI could also be attributed to the presence of dietary fibre in all four of the read samples, as well as in the standard wholemeal bread. The lower GI of the SWM bread compared to the other standard samples is due to the higher fibre content in this bread sample than in white bread; fibre remains undigested in the upper GIT, which results in low glucose release.

### 3.2. Study 2: Acceptability in School Breakfast Clubs

In School 1, most/all of the children attending ate breakfast at the club. In School 2, only about half of the ~35 children attending ate breakfast at the club. However, on some intervention days, some children opted to try the novel foods, especially the cereals and cut fruit. The distribution of extra food and fibre eaten during the intervention was quite different between the schools. In School 1, the extra fibre chosen primarily reflected the fact that each child was eating more food overall, including high-fibre intervention foods. In School 2, this effect was also present, but some increases in foods chosen were driven by children eating who did not usually eat. Some children made multiple visits to the breakfast buffet. We report the average food choices in terms of their contribution to the total fibre chosen per child present at the breakfast club. For School 2, this presentation has the effect of reducing the amount per child of food/fibre chosen in a typical breakfast.

The fibre chosen represents the upper limit of the group’s fibre intake; the fibre consumed will be a certain % of this, with uneaten/wastage food accounted for. For example, children often did not eat bread crusts. Three intervention foods (Strawberry and Apple Rice Krispie Shapes, Augmented Cereals, and Whole Fruit) were noted as generating relatively high wastage. The fibre content of both Whole Fruit and Cut Fruit were estimated as 1.5 g/100 g, based on a conservative estimate given the fibre content of the various fruits offered.

On average, over the 12 days on which we offered intervention foods as additions to the existing Magic Breakfast range, fibre consumption increased significantly (*p* = 0.0024, CI = 0.95, paired *t*-test) from the mean at baseline (1.87 g), by 1.99 g to 3.86 g per child present. See Table 1 for the details of overall fibre chosen (in g/child present) over the course of the six research weeks in the two schools. We present a more detailed results breakdown below to assess liking and take-up, and hence feasibility, for the higher-fibre breakfast menus, but not to demonstrate increases in fibre consumption resulting from any given product or food type, for three reasons:

1. Magic Breakfast’s standard range was a relatively high-fibre baseline, reflecting their deliberate efforts to increase the fibre density of their offer. The intervention foods therefore often replaced alternatives with an already-high fibre content.
2. The baseline variability was relatively high between research days (primarily but not exclusively due to presence/absence of bagels), so individual comparisons may be misleading.
3. There were some quite clear (positive) novelty effects. This bodes well for introducing novel foods to breakfast clubs, but also adds a confounding factor to our results.

#### 3.2.1. Breads/Toast with Plant-Based Spread

**Figure 7:**
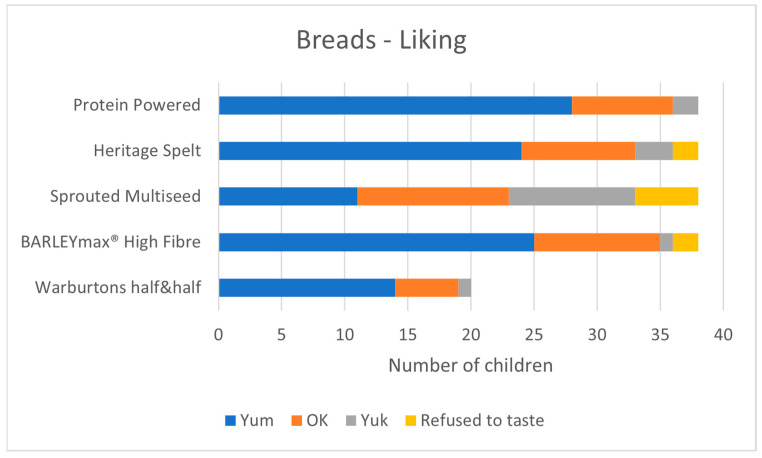
Child liking for the four Prograins bread products, plus the 50% bread served as standard.

The Warburton’s half&half ‘50%’ bread, recently introduced as the standard by Magic Breakfast, appeared to be widely accepted by the children. In a prior pilot study, we conducted taste-and-rate sessions using this 50% bread, and we include these results in Figure 7. This bread was liked by most children, to a similar degree as the more popular intervention breads. Notably, though, white bagels were more popular when (sporadically) available.

Taste-and-rate activity: Just over half (38/69) of the children present at the breakfast clubs chose to take part in the taste-and-rate activity. The others preferred to play and talk with their friends. The intervention breads were liked by many children. The Sprouted Seed bread was the least popular (29% Yum, 60% Yum or OK). The other three breads were liked to a similar degree (collectively 67% Yum, 94% Yum or OK) as the 50% bread which served as a standard (70% Yum, 95% Yum or OK). See Figure 7.

Breakfast Service: Liking in the taste-and-rate was broadly reflected in the uptake of the intervention breads during breakfast service. At baseline, with only Warburton’s 50% bread on offer, a mean of 0.49 slices (per child) were chosen. When the intervention breads were available, this dropped to 0.31 slices, whilst children chose a mean of 0.77 slices (per child) of the intervention breads. Of the latter, the children chose 32% Heritage Spelt, 30% Protein Powered, 20% BARLEYmax^®^, and 17% Sprouted Multiseed. Notably, the more ‘bitty’ breads (Sprouted MultiSeed and BARLEYmax^®^) were chosen less frequently, highlighting the importance of both visual and tactile texture.

No notable change in wastage was observed. The main wastage for toast was unwanted crusts, for all bread types. These data indicate that many children liked and chose the intervention breads. The patterns were quite different in the two schools, so we display them separately here. Note that the intervention foods (aways at the bottom in these cumulative graphs) were only offered on Day 1 and Day 2 (so appear as zero on Day 0/baseline, when they were not offered).

**Figure 8:**
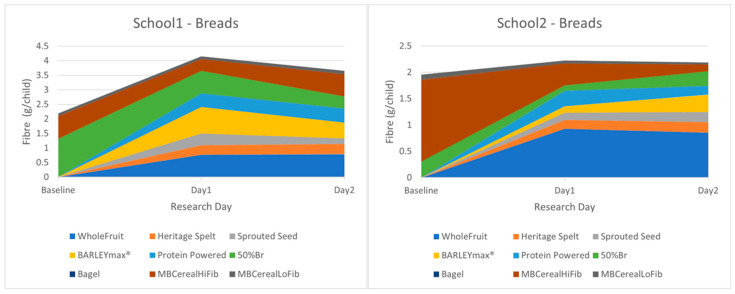
Breakfast choices in School 1 and School 2 with novel Prograins breads offered. MBCereal indicates cereals served as standard in Magic Breakfast clubs, with high (HiFib), or low (LoFib) fibre.

In School 1, 50% toast was very popular, and made up most of the fibre consumed at baseline, probably because this school offered honey and jam with toast. When offered the intervention breads, the children consumed a lot, boosting both fibre density and overall quantity chosen, leading to an overall increase in fibre consumption which was further increased by the additional choice of Whole Fruit. In School 2, the high-fibre cereals (Weetabix and CrunchyBran) in the standard Magic Breakfast range were the most popular foods at baseline. During intervention days, many children swapped their cereal for intervention bread/toast. As the cereals (especially Crunchy Bran) were quite a lot higher in fibre per portion than most of the intervention breads, this resulted in a fall in fibre consumption from grain-based foods, but this was compensated for by Whole Fruit, such that overall fibre consumption remained similar. See Figure 8.

#### 3.2.2. Ready-to-Eat Breakfast Cereals (With Optional Milk)

The standard Magic Breakfast cereal offer includes a range of six higher-fibre cereals and two lower-fibre cereals. These were not all offered consistently in the breakfast clubs. By a large margin, the most popular cereals in the standard Magic Breakfast range were Weetabix and Crunchy Bran (both higher-fibre options), followed to a lesser extent by porridge (also high-fibre) and Cornflakes and Rice Krispies (both low-fibre). Thus, cereal choice was already dominated by higher-fibre cereals at baseline.

**Figure 9:**
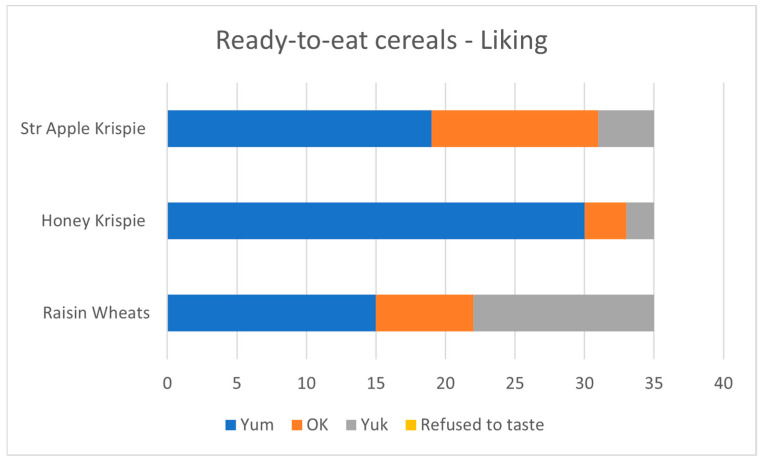
Children’s liking for the three intervention cereals.

Taste-and-rate: Thirty-five children opted into the taste-and-rate activity. The Honey Rice Krispie shapes were widely liked (86% yum). The Strawberry and Apple flavour was somewhat less well liked (60% yum), with a few children expressing particular dislike. The Raisin Wheats were least popular (43% yum). See Figure 9.

**Figure 10:**
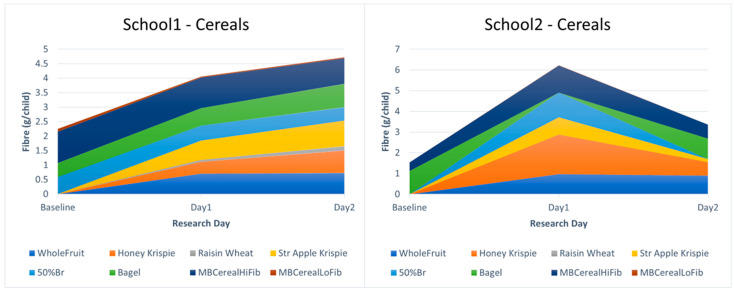
Breakfast choices in School 1 and School 2 with novel high-fibre cereals offered. MBCereal indicates cereals served as standard in Magic Breakfast clubs, with high (HiFib), or low (LoFib) fibre.

Breakfast choices: The liking measures were reflected in the breakfast choices. At baseline, a mean of 0.16 portions of high-fibre cereal from the standard Magic Breakfast range were chosen, and a mean 0.07 portions of low-fibre cereal. This baseline cereal take-up was relatively low compared to some other baseline days, apparently due to the presence of bagels on that day. During breakfast service, the Honey Krispie Shapes were popular (mean 0.26 portions per child), and the RaisinWheats were not (mean 0.02 portions per child). The Strawberry and Apple Krispie Shapes were quite popular by choice (mean 0.16 portions per child), but extra wastage was noted for this cereal, whose flavour was expressively disliked by some children. In School 1, the increase in fibre chosen per child on the intervention days (Day 1—1.8 g, Day 2—2.47 g) was driven primarily by children having a bowl of intervention cereal on top of their usual breakfast, plus Whole Fruit. In School 2, the steep Day 1 rise in fibre chosen per child present (up 4.7 g) was driven primarily by children who usually did not eat breakfast at the breakfast club, but who did eat on Day 1, when the intervention Cereals and Whole Fruit were first available. On Day 2, the novelty seemed to have worn off, and most of the usual non-eaters returned to not eating breakfast at school, such that the increased fibre chosen relative to baseline was lower, at 1.8 g/child, more in line with results observed on other days. See Figure 10. Overall, our results indicate that higher-fibre cereals are a feasible option for primary school breakfast clubs. Indeed, this approach has been adopted by Magic Breakfast, and from our observations, this effort is proving successful. The apparent novelty effect, where usual non-eaters became eaters because they wanted to taste the novel foods, somewhat distorts the data here, but it is encouraging in that enthusiasm for novel foods would likely help with introducing novel foods into breakfast club settings.

#### 3.2.3. Low-Fibre Cereals Augmented with Strawberries and Porridge Oats

Our final intervention food was aimed at children who are resistant to eating higher-fibre RTEC cereals. To reach these children with a higher-fibre breakfast option, we added fruit (strawberries) and porridge oats to the low-fibre cereals served in Magic Breakfast clubs (Rice Krispies and Cornflakes cereals), to augment their fibre content. Also, we changed the fruit offer from Whole Fruit (bananas, apples, satsumas, grapes) to a platter of Cut Fruit, which included watermelon, melon, apples, bananas, grapes, grapefruit, and strawberries.

**Figure 11:**
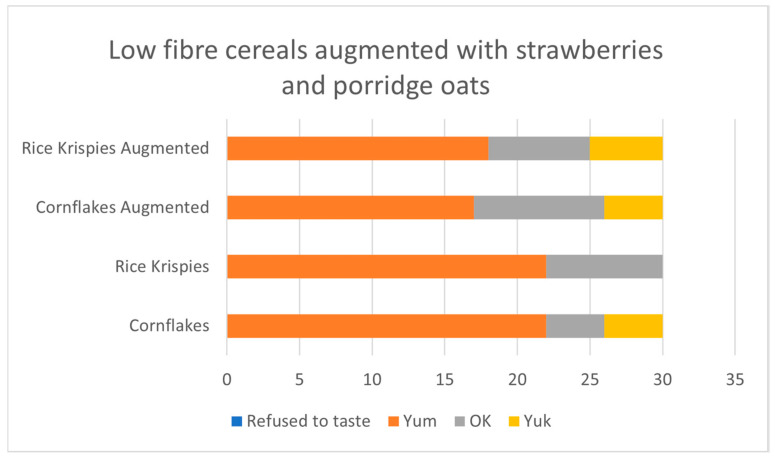
Children’s liking for low-fibre cereals augmented with strawberries and porridge oats.

Taste-and-rate: Thirty children opted into the taste-and-rate activity. The augmented cereals were slightly less liked than the cereals alone, but the augmented version remained quite well liked overall. See Figure 11.

**Figure 12:**
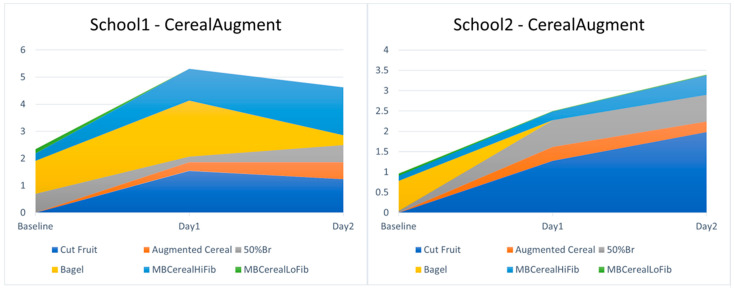
Breakfast choices in each school with augmented low-fibre cereals offered. MBCereal indicates cereals served as standard in Magic Breakfast clubs, with high (HiFib), or low (LoFib) fibre.

Breakfast service: The augmented cereals did largely replace the choice of low-fibre cereals alone, which was their intended function. At baseline, 11 portions of low-fibre cereal were chosen (0.16 portions/child). Over the four intervention days (in two schools) during which the augmented cereals were offered, only 2 portions of low-fibre cereal alone were chosen (0.018 portions/child), versus 20 portions (0.18 portions/child) of augmented cereal chosen. However, researchers noted higher than usual wastage of this intervention food, apparently driven by children attracted by the strawberries, but disliking the porridge oats. The augmented cereals might be more worthwhile in breakfast clubs where low-fibre cereals are consumed in higher amounts, but made a small impact here. The Cut Fruit, however, was very popular, and was the main driver for increasing fibre chosen on intervention days (mean 1.5 g/child), driving an overall increase in fibre chosen on intervention days. Wastage was noted to be lower for Cut Fruit than for Whole Fruit (children would quite often eat half an apple or banana and leave the rest, given Whole Fruit). See Figure 12.

## 4. Discussion

In Study 1, the Prograins breads all presented more desirable glucose release curves during in vitro digestion than the standard white bread, 50%, and wholemeal breads. The Sprouted Multiseed bread exhibited the most desirable (i.e., shallowest) curve. However, this darker, seeded bread was liked by the fewest children. The Protein Powered bread had a low initial spike, a relatively sustained glucose supply, a relatively low 2 h GI, and the lowest overall GI. Importantly, most children liked it well. The very high fibre content of the BARLEYmax^®^ bread did not translate into a marked difference in the shape of the glucose release curve compared to the other Prograins breads. Its visible ‘bits’ seemed to put some children off choosing it, but amongst children who did taste it, it was well liked. The Heritage Spelt bread has the lowest fibre of all the Prograins breads, with a similar level to the standard 50% bread. Whilst it yielded a relatively high 2 h and overall GI, its glucose release curve exhibited a desirable, steady profile of glucose release, second only to Sprouted Multiseed for continued glucose release after two hours. Grain species effects on glucose release have been noted previously [29].

Dietary fibre (DF) changed the microstructure of the bread (i.e., produced a network able to entrap starch granules, thus protecting them from amylolytic enzymes); DF can also alter the viscosity of the chyme, which may reduce the digestibility of the bread samples in the oral and gastric phases, leading to a lower GI [39]. Similar effects were observed with the high-protein samples, where the presence of a high starch content would result in the formation of starch–protein complexes, which would restrict enzymatic hydrolysis during digestion [40]. Secondly, a higher lipid content in the SWM bread sample could also have affected the GI, because lipid can coat starch granules, thus preventing the access of amylases to their substrate [37]. Thus, a range of factors influenced the shape of the glucose release curve; fibre, protein, grain species, seeds, and particulate persistence. Overall, the Prograins breads achieved large improvements in glucose release curves via a range of ingredients and interactions relative to the ‘standard white bread’, and small improvements relative to the standard wholemeal bread. Further research should confirm these results in vivo on glucose metabolism (e.g., insulin response), and examine potential wider effects on fibre-related health outcomes (e.g., on gut microbiome).

Study 2 found that the children had varied individual preferences, and different choice patterns were also observed at each breakfast club. Overall, children liked and chose the intervention foods in considerable quantity, with some products notably preferred over others. A variety of higher-fibre foods will likely be necessary to cover the range of preferences and eating habits. Introductory taste-and-rate sessions for novel foods could both help to familiarise children and empower them in their decision making, and inform school staff and providers which products are likely to be most successful in their setting. However, controlled studies would be required to properly understand their utility, efficacy, and importance for outcomes. This pattern of results suggests that if a single bread is provided in breakfast clubs, then a smooth/non-seeded bread with a lighter visual aspect will be the most universally accepted, which is compatible with existing evidence regarding children’s preferences [15]. However, if two or more bread options are provided, then darker and seeded breads are viable options for a significant minority of children. The children’s preference for bagels was notable, and bagels were also distributed beyond the breakfast club to other children arriving at school. Staff and children explained that bagels held their texture and sensory appeal better than toast, especially on becoming cold. Bagels were also less wasted, due to children not leaving crusts, as many did for the loaf breads. This suggests that a bagel product with a smooth texture and light visual aspect, but high fibre and perhaps protein content, could effectively combine digestive health benefits with wide acceptability and broad distribution across the pupil population.

Breakfast cereals comprise the main element of breakfast for many children [8], but breakfast cereals aimed at children have tended to be low in fibre and high in sugar historically, contributing to the fibre gap [41]. The Rice Krispies Shapes incorporate inulin to increase both fibre content and non-sugar sweetness [42], and were quite popular with the children. Crunchy Bran and Weetabix (offered as standard by Magic Breakfast) are not expressly aimed at children, but were the most popular cereals in the Magic Breakfast offer. It therefore appears viable for school breakfast clubs to offer children a menu comprising only higher-fibre cereal products, especially as the range of such products grows. Whole Fruit was moderately popular (grapes were the most popular fruit), but generated quite a lot of waste from half-eaten pieces of fruit. Cut Fruit was very popular and generated less waste. Given the fibre and wider health benefits of consuming a wide variety of fresh fruit [43], and the prevalence of low fruit consumption amongst children from low-SES backgrounds [2], the provision of a wide variety of good-quality cut fruit in primary school breakfast clubs would be desirable, and is likely to be well accepted by children. The primary barriers are likely the high cost of good-quality fruit, the logistical challenges of supplying fresh produce, and the time required by staff to cut up the fruit.

## 5. Conclusions

In a 2020 review, five main factors for facilitating wholegrain intake were identified: (i) the availability and variety of foods, (ii) sensory appeal, (iii) purchase cost, (iv) a familiarisation period, and (v) communication and labelling [44]. Our approach and findings accord well with the first four. Providing a variety of well-designed, high-fibre food products suited to children was effective, the school setting insulates children/parents from purchase costs, and the taste-and-rate activity provided a familiarisation step. Although cost is an issue for policy makers, the cost-benefit analysis s of improving school child nutrition is positive [7], and prevention-first approaches are an explicit aim of the current government [45]. Whilst limited to a small sample, our results suggest that children in school breakfast clubs will like and choose higher-fibre foods. Our key finding is that shifting breakfast provision towards higher-fibre breads and other breakfast foods in breakfast clubs was feasible in our sample. Larger-scale breakfast club interventions limiting options to only foods which are a good source of fibre are a focus of our ongoing work, and look likely to be successful from preliminary results. Universal school breakfast trials planned by the UK government for 2025 would be an excellent opportunity for larger trials of this choice architecture constraint, with a view to ensuring that universal breakfast provision improves on the currently low fibre consumption in children and the healthiness of the national diet in the long term.

## Figures and Tables

**Table 1:** Fibre chosen at baseline and during interventions over six intervention weeks in two schools.

Mean Fibre Chosen (g/Child Present)	Baseline	Day 1 Total	Day 2 Total	Day 1 and 2	Mean Change
Week 1, School 1	2.19	4.15	3.66	3.905	1.715
Week 1, School 2	1.95	2.22	2.19	2.205	0.255
Week 2, School 1	2.25	4.06	4.73	4.395	2.145
Week 2, School 2	1.54	6.22	3.36	4.79	3.25
Week 3, School 1	2.34	5.31	4.62	4.965	2.625
Week 3, School 2	0.96	2.5	3.39	2.945	1.985
Average (mean)	1.872	4.077	3.66	3.867	1.995

## Data Availability

Anonymised data will be made openly available via the University of Leeds Library repository.

## Supplementary Materials

The following supporting information can be downloaded at https://www.mdpi.com/article/10.3390/nu17020308/s1: Infogest protocol as followed in this study Table S1: Semi-dynamic digestion parameter to start the process; Table S2: Composition of each simulated digestive fluid at a concentration of 1.25×.
