# Supplementary material for: Novel Fibre-Rich Breads Yield Improved Glucose Release Curves and Are Well Accepted by Children in Primary School Breakfast Clubs
Source: Nutrients. 2025 Jan 16;17(2):308. doi: 10.3390/nu17020308 (PMC11767593; doi:10.3390/nu17020308)
Supplement: Supplementary file 1 [file nutrients-17-00308-s001.zip › nutrients-3398587-supplementary.pdf]

Novel fibre-rich breads yield improved glucose release curves and are well accepted by children in primary school breakfast clubs

Supplementary materials

S1.1 The intestinal phase was performed separately on each gastric emptied aliquot using the static version of the INFOGEST protocol. Parameters used in the semi-dynamic model to start the digestion are given in table S1.

Table S1: Semi-dynamic digestion parameter to start the process.

		Barley max	*Heritage spelt*	*Protein power*	*Sprouted multispeed*	*Standard White bread*
Food Composition (Kcal/mL)	Lipid	0.144	0.162	0.162	0.639	0.108
	Protein	0.432	0.464	0.528	0.456	0.348
	Carbohydrate	1.528	1.66	1.6124	1.484	1.78
	Total Calories	2.104	2.286	2.3024	2.579	2.236
	Dry weigh (%)	64.15	65.65	59.8	66.8	59.5
Starting quantity of Meal						
	Quantity of Food (g)	20	20	20	20	20
	Dry Weight of Food(mL)	12.83	13.13	11.96	13.36	11.9
Gastric digestion time (min)		105.20	114.30	115.12	128.98	111.80
Gastric emptying points		5	5	5	5	5

The electrolyte stock solutions of digestion fluids (x1.25 concentrated), including electrolyte simulated salivary fluid (SSF), electrolyte simulated gastric fluid SGF and electrolyte simulated intestinal fluid (SIF) were prepared according to Brodkorb et al. (2019) [34], pH was adjusted to 7 by 1M HCl and stored at −20 °C. Composition of each electrolyte solution is given in table S2.

S1.2 Static *in vitro* oral digestion

In the reaction vessel, which was a v-form vessel (Yorlab, UK) with a thermostat jacket (37 °C), 20 g of minced bread samples (Solid foods need to be chewed, which is simulated by mincing a piece of bread in manual mincer) were mixed with the simulated oral solution consisting of SSF, $CaCl_2(H_2O)_2$ (0.3 M) and milli-Q water to a final ratio of 1:1 with the dry weight of food (Table 1). The volume of oral mixture solution added varied according to the dry weight of each bread sample. The pH of the mixture was adjusted to 7. After 2 minutes of mixture agitation (which was left at 37 °C with continuous agitation at 100 rpm using an overhead stirrer (Hei-TORQUE, Germany) with a 3D printed stirrer paddle) α-amylase (150 U/mL in SSF) was added to the mixture (food + SSF) and oral phase was allowed to continue for 3 more minutes.

Table S2: Composition of each simulated digestive fluid at a concentration of 1.25×

			SSF *(make up to 400 mL)*		***SGF*** *(make up to 400 mL)*		***SIF*** *(make up to 400 mL)*	
			pH 7		pH 7		pH 7	
Constituent (Mw, Da)	**Stock conc. (g/L)**	**(M)**	**Vol. of stock (mL)**	**Conc. in SSF (mM)**	**Vol. of stock (mL)**	**Conc. in SGF (mM)**	**Vol. of stock (mL)**	**Conc. in SIF (mM)**
***KCl** (74.55)*	37.3 (1.87 g in 50 mL)	0.5	15.1	15.1	6.9	6.9	6.8	6.8
***KH_2PO_4** (136.09)*	68 (0.68 g in 10 mL)	0.5	3.7	3.7	0.9	0.9	0.8	0.8
***$NaHCO_3$** (84.007)*	84 (8.4 g in 100 mL)	1	6.8	13.6	12.5	25	42.5	85
***NaCl** (58.44)*	117 (2.92 g in 25 mL)	2	0	0	11.8	47.2	9.6	38.4
***$MgCl_2(H_2O)_6$** (203.30)*	30.5 (0.15 g in 5 mL)	0.15	0.5	0.15	0.4	0.1	1.1	0.33
***$(NH_4)_2CO_3$** (96.09)*	48 (0.24 g in 5 mL)	0.5	0.06	0.06	0.5	0.5	0	0

S1.3 Semi-dynamic *in vitro* gastric digestion

A gastric mixture solution was prepared to have a final ratio of 1:1 with the oral bolus (food, SSF, CaCl2(H2O)2, milli-Q water and α-amylase). The gastric mixture solution consisted of SGF (SGF, CaCl2(H2O)2 (0.3 M) and milli-Q water), HCl (1 M) and pepsin solution (4000 U/mL of gastric mixture solution). The pH of the oral bolus was decreased to simulate the fasted state in the stomach by adding 10% of the SGF solution and HCl until it reached pH 2. The remaining 90% of SGF solution, pepsin solution (4000 U/mL of gastric mixture solution) and HCl (1 M) was added continuously during the course of gastric digestion using an automated dosing device (800 Dosino, Metrohm, Switzerland) with an automatic titrator (902 Titrando, Metrohm, Switzerland). The enzyme solution was delivered by a syringe pump (Cole-Parmer, USA). The rate at which these solutions were delivered was dependent on the total gastric digestion time for each bread-meal (Table 1). The gastric content was mixed at 10 rpm (using the same overhead stirrer as in the oral phase) and 37 °C during the total gastric phase time determined for each bread sample. The gastric residence time was based on the energy density of the bread samples and gave an emptying rate equivalent of 2 kcal/minute. Gastric emptying (GE) was simulated by taking five aliquots, referred to as GE1-5, corresponding to the portion of digested bread-meals that would be delivered into the duodenum. For each GE point, a sample was taken manually at the calculated time from the bottom of the vessel by using a 10 mL pipette tip and the pepsin activity in each aliquot was stopped by adding 2M NaOH to increase the pH above 7. These GE aliquots were snap-frozen in liquid nitrogen and stored at −80 °C for subsequent *in vitro* small intestinal digestion.

S1.4 Intestinal phase

The intestinal phase digestion was done according to the INFOGEST static digestion protocol separately on each GE aliquot emptied from the gastric phase. Each GE aliquot was mixed with intestinal fluids (SIF) in 1:1 (v/v). SIF includes eSIF, pancreatin enzyme solution (trypsin activity: 200 U mL^{-1} in SIF), bile (20mmol/L SIF), water, $CaCl_2(H_2O)_2$ and 1M NaOH. pH of the intestinal digestion mixture was adjusted to 7 where required. Finally, the mixture was placed in the shaking water bath at 37 °C (100 rpm) for 2 hours.
